# Debating the ‘integration of Islam’: the discourse between governmental actors and Islamic representatives in Germany and the Netherlands

**DOI:** 10.1186/s40878-018-0086-2

**Published:** 2018-08-02

**Authors:** Matthias Kortmann

**Affiliations:** Faculty of Humanities and Theology, Technical University of Dortmund, Emil-Figge-Str. 50, 44227 Dortmund, Germany

**Keywords:** Political opportunity structures, Institutionalism, Constructivism, Framing, Integration of Islam, Religious governance

## Abstract

This paper examines what influences the views of governmental and Islamic actors in consultations on the integration of Islam in Germany and the Netherlands. Disentangling institutionalist and constructivist assumptions within the concept of political opportunity structures and employing a content analysis of primary documents and interviews, the paper shows that expectations of both approaches apply: On the one hand, Islamic organizations (as challengers) and governmental representatives (as defenders of the status quo) each problematize the issue differently. Yet, their views also depend on specific national contextual factors (i.e. regimes of immigrant integration and religious governance) and, therefore, differ cross-nationally, too. The paper argues that it is fruitful to uncover the ways participants in the discourses define and conceive of central terms and concepts prevalent in order to disclose their fundamental motivations, interests, and strategies.

## Introduction

After decades of immigration from countries with high population shares of Muslims to Western European countries, the integration of Muslims has become a central political issue. In recent years, many governments in receiving countries have established advisory boards to enable consultations with Muslims on issues related to the *integration of Islam*. A growing number of studies has focused on the structures and participants of these consultations as well as on the negotiations. Particularly comparative studies have tended to choose an institutionalist approach trying to uncover nationally divergent opportunity structures (cf. Dolezal, Helbling, & Hutter, 2010; Fetzer & Soper, 2005; Koopmans & Statham, 2000; Koopmans, Staham, Giugni, & Passy, 2005). However, at a closer look, the political opportunity structures (POS) approach also contains assumptions of the – rather constructivist – framing approach. This is striking since the two approaches allow for somewhat contradictory expectations. From an institutionalist point of view, one might expect actors to use cross-nationally divergent argumentative patterns assuming that national frameworks provide a focal point for both governmental representatives and civil society groups when expressing their views. Consequently, with regard to the study at hand it might be expected that views on the *integration of Islam* can be traced back to particular national contextual factors which both the respective governmental and Islamic actors tend to adapt their positions to. In contrast, constructivist researchers would rather suggest that views of governmental actors, which usually try to defend a status quo, are likely to differ from those of organized societal interests which rather act as challengers. Researchers that use explicitly constructivist perspectives mostly pursue single country studies in order to uncover perceptions and framing strategies of governmental and/or Islamic representatives (Halm, 2013; Mourão Permoser, Rosenberger, & Stoeckl, 2010; Schiffauer, 2003; Teszan, 2012). Furthermore, studies have scarcely explicitly applied and evaluated the significance of both institutionalist and constructivist approaches. This is where this study wants to tie in: Applying a comparative approach, it examines views of both governmental actors and Islamic organizations in consultations on the *integration of Islam* in Germany and the Netherlands. Before doing so, it tries to disentangle institutionalist and constructivist expectations within the POS in order to better make use of them for answering two connected guiding questions: To what extent do the views of governmental actors and Islamic organizations on the *integration of Islam* depend on their respective status as defenders and challengers of a status quo? And in what way are their positions influenced by national contextual factors they are confronted with?

Most of the studies mentioned have analysed (power) structures of negotiation contexts as well as actors involved and their respective claims when examining debates between governmental and Islamic representatives on the *integration of Islam*. Furthermore, studies have also shown which issues have been emphasized by both sides and what preconditions for a successful integration have been perceived. However, studies have rather neglected underlying definitions of fundamental terms and concepts such as *religion in the public sphere*, *religion* vs. *state*, *religious pluralism* or *Islam*. Yet, it is not least these definitions and perceptions that help to comprehend (differences between) the positions of actors involved in the debates and, consequently, to explain why discourses proceed the way they do and produce the results they produce. Therefore, this paper wants to uncover how governmental and Islamic representatives in Germany and the Netherlands each define or (strategically) frame the *integration of Islam*.

The rest of this paper is structured as follows: First, institutionalist and constructivist assumptions with regard to the discourse between governmental and Islamic representatives on the *integration of Islam* will be disentangled. Subsequently, the methodological approach of the study, thus the qualitative discourse analysis is presented. Third, the relevant factors of the national institutional contexts will be considered – that is, national regimes of immigrant integration and religious governance – as possible sources of national POS. Fourth, governmental and Islamic actors involved in the discourse will be introduced before the empirical results of the analysis will be provided in section five. Finally, conclusions concerning the research questions will be drawn (section 6), and implications of the scientific and political debate on the *integration of Islam* will be discussed (section 7).

## The political opportunity structures and the framing approaches: institutionalist and constructivist assumptions

As an institutionalist approach, the concept of POS expects an impact of contextual factors on activities and strategies of political actors which operate within the respective political context. Therefore, although the POS approach was inspired by studies of protest behaviour (Eisinger, 1973)^1^ and developed in analyses of emerging social movements, its assumptions can also be referred to other political players such as governmental actors. In his book *Power in Movement*, Sidney Tarrow defines POS as ‘consistent – but not necessarily formal or permanent – dimensions of the political environment that provide incentives for people to undertake collective action by affecting their expectations for success and failure’ (Tarrow, 1994, p. 85). Thus, political opportunities may come about not only through stable institutional structures but also through informal and volatile political procedures. Analysing new social movements in Western Europe, Kriesi (2004, p. 67) particularly refers to dominant strategies, thus ‘procedures typically employed by members of the political system’ within political processes.

Although the formal and informal context is important for political processes in general, ‘opportunity structures can vary enormously from one issue field to another’ (Koopmans et al., 2005, p. 19). In political negotiations, political actors thus (more or less consciously) adapt their strategies and argumentative patterns to opportunities and constraints derived from stable or volatile formal, informal, and discursive opportunity structures.

Fetzer and Soper (2005) argue that not only expectations formulated by the state but also claims by Islamic organizations in Germany, France, and Great Britain reflect opportunity structures such as regimes of religious governance prevalent in the respective countries. Focusing on the same countries, Brunn (2012, p. 284) uncovers nationally different ‘manifestations of the independent variable “combination between religion and integration policy”’ which influences the way the issue of integration is dealt with.

Relating these results to the study presented in this paper, one might expect arguments of the governmental and Islamic representatives in Germany to differ from arguments of their counterparts in the Netherlands since we find diverging ‘issue-specific opportunity structures’ (Dolezal et al., 2010) in the two countries when it comes to regimes of immigrant integration as well as religious governance (Expectation 1).

However, the POS approach also includes assumptions that point to likely differences between governmental actors and interest groups when acting as political players. Koopmans and Statham (2000) argue that it is indeed both sides that are aware of and respond to opportunity structures, but it is the latter contingent for whom such structures are most important because interest groups depend on material and immaterial resources and must therefore grasp the opportunities available to them. One resource in particular can be seen as the most fundamental: legitimacy. By presenting themselves and their goals as being in accord with the society’s shared values, expectations and basic rules and regulations, interest groups can ensure that their issues and goals resonate with the experiences, interests, values, and perceptions of governmental representatives, other societal actors and a larger portion of the population as a whole. In this context, the framing perspective becomes significant. Based on assumptions of symbolic interactionism and constructivism, the framing perspective highlights the way issues are perceived in public discourses (Koopmans & Statham, 2000, p. 35). Zald (1996, p. 262) defines frames as ‘specific metaphors, symbolic representations, and cognitive cues used to render or cast behaviour and events in an evaluative mode and to suggest alternative modes of action’. The process of framing can be described as a ‘competition among perspectives describing the same underlying phenomenon’ (Baumgartner, Berry, Hojnacki, Kimball, & Leech, 2009, p. 166) that takes place between the opposing sides in a political discourse. In this competition, the opponents use frames as a way of highlighting and interpreting problems and then pointing to appropriate ways of handling or resolving them (Zald, 1996, p. 265). Those who challenge the status quo need to find ways of adapting their frames to the ‘hegemonic discourses’ (Koopmans & Statham, 2000, p. 36) in order to strengthen the legitimacy of their demands. In addition, framing is crucial for interest groups because it serves as a means of gaining the attention of the public and governmental actors. Zald argues that ‘activists in movements and countermovements have a stake in developing metaphors, images, and definitions of the situation that support alternative programs’ mostly directed toward abolishing injustice in any form (Zald, 1996, pp. 266-267).

Consequently, these assumptions suggest that Islamic organizations, wishing to have the same standing as the Christian churches, will challenge the status quo and thus apply argumentative patterns that differ from those used by governmental representatives who tend to support the status quo, thus the prominent status of (organized) Christianity. This assumption is also supported in a study by Mourão Permoser et al. (2010) on the dialogue between the Austrian government and the Islamic Religious Community in Austria (*Islamische Glaubengemeinschaft in Österreich*, IGGiÖ), the officially recognised Islamic contact partner. According to the authors, representatives from the IGGiÖ believe the dialogue to be about ‘the governance of religious diversity’ – against the background of their wish of Islam being treated equally as other religions and Christianity in particular. However, governmental representatives locate these consultations in the context of the ‘integration of immigrants’, which rather implies to treat the organized religion of Islam – as an immigrated religion – differently from the churches which represent the domestic religion of Christianity. This is also in line with Teszan’s (2012) impression when analysing consultations between Islamic organizations and the government in Germany. Teszan argues that German officials have less focused on integrating Islam structurally in the same way as Christianity and Jewry (and as hoped for by the Islamic organizations). Instead, their aim has been to develop a ‘social contract’ (Teszan, 2012, p. 45) with all Muslims living in Germany in order to enhance the societal integration of this minority group (a point which is also made by Brunn, 2012). Consequently, according to this, the central frame used by Islamic organizations is likely to be the one of *equal treatment* whereas government representatives will rather refer to the frame of *treating unequal things unequally* (expectation 2).

Contrasting the two expectations developed above, the question arises which of the arguments might prevail within consultations between Islamic organizations and governmental representatives in Germany and the Netherlands: Do Islamic (as challengers) and governmental actors (as supporters of the status quo) argue differently because of their divergent roles the play in the negotiations? Or do Islamic representatives ‘incorporate receiving-society issues into their own positions’ (as suggested by Schiffauer (2003, p. 153) with regard to Islamic organizations in Germany) against the background of their ‘strong needs to be trusted’ (Mattes, 2017, p. 51) which prompts them to pursue the strategy of ‘discursive assimilation’ (Schiffauer, 2003, p. 156)?

## Methodology: qualitative discourse analysis

The general approach the paper pursues is a qualitative discourse analysis which focuses on the meaning, interpretation and political-discursive constitution of reality. On the one hand, the discourse analysis tried to identify the *equal treatment* and *treating unequal things unequally* frames as well as frames that can be traced back to nationally specific opportunity structures in Germany and the Netherlands. On the other hand, interpretative patterns used by involved actors were also uncovered inductively. In this context, statements by representatives of the Dutch and German governments and those of Islamic organizations on the *integration of Islam* in the two countries were analysed by means of qualitative content analysis (Keller, 2004; cf. Mayring, 2000). Sources included statements of involved actors in the media, via press releases, in position papers, in speeches or in interviews. The research project made use of 15 semi-structured interviews that were conducted in 2008 with representatives of (Turkish-)Islamic organizations in both countries which were directly included in negotiations within the respective advisory boards (see chapter IV). Furthermore, statements published on the organizations’ websites or in press releases during the time period of the analysis (2006 to 2012) were included. Governmental representatives that were accounted for were those particularly responsible for the negotiations within the advisory boards (Ministers of the Interior in Germany and Ministers of Integration as well as Justice in the Netherlands). Furthermore, position papers released by the governments on issues touching the *integration of Islam* were examined.

## Germany vs. the Netherlands: national regimes of immigrant integration and religious governance

Until recently, the Netherlands was perceived as a typical representative of a multiculturalist integration policy, encouraging minority groups to preserve their ethnic identity and naturalizing immigrants rather quickly. Contrary to this, the German concept of citizenship policy was described as an ethnic concept since naturalization for persons with non-German parents was rather difficult (Koopmans et al., 2005).

Although the concepts of citizenship of the two countries have become more similar in recent years, their respective regimes of immigrant integration still provide different opportunities when it comes to the political participation of ethnic minorities. In the Netherlands, regular meetings between the Minister of Integration and representatives of various advisory boards from different minorities are regulated by the Law on Minority Consultation (*Wet Overleg Minderhedenbeleid*, WOM). The WOM instructs these self-promoting organizations to unite in ethnically based cooperative associations (*inspraakorganen*) which are subsidized by the government (Musch, 2011).

In Germany, there is no comparable legal basis for funding and working with migrant organizations. Non-nationals’ rights to participation are only granted at the *individual* and not the *collective* level. Recently, however, migrant organizations which had been regarded with suspicion before have been consulted more frequently by political actors of all federal levels to address questions of integrating migrants. In 2006 the so-called Integration Summit (*Integrationsgipfel*) was established. Contrary to its Dutch counterpart, the National Consultation on Minorities (*Landelijk Overleg Minderheden*, LOM), the Integration Summit does not have established structures, and the participating migrant organizations are not provided with subsidies.

When it comes to the regime of religious governance, the German constitution explicitly emphasises the role of religious communities in public life.^2^ It establishes the legal status of both religious communities (Article 7) and public corporations (Article 140) for religious organizations. Whereas the Christian Churches held both statuses well before the German constitution was ratified, other religious communities must apply for them. If religious organizations want to offer religious education in public schools, they need to be recognised as religious communities by the federal states responsible in this matter (Article 7[3] GG). Religious organizations that are recognised as public corporations have the right to levy a church tax on their members, which is administrated by government tax authorities (Campenhausen & de Wall, 2006, pp. 256-287). In addition, the German government bears most of the expenses for the personnel of the social welfare agencies that the churches have established on their own. Religious communities that are recognised as public corporations can also serve on several advisory boards because they are perceived to be ‘socially important groups’ (Leggewie, 2003, p. 174).^3^ Again, the German federal states are responsible for granting this recognition. Although religious communities in the Netherlands cannot obtain similar public recognition, the Dutch government has provided opportunities for religious organizations as a means of establishing religious pluralism in the country. This includes the right to establish religious broadcasting corporations and denominational schools (Bijsterveld, 2005, p. 399).

For Islamic organizations, the most important differences between the German and Dutch regimes of religious governance can be seen in the matter of official recognition. In April 2007, the four largest Muslim organizations established the Coordination Council of Muslims in Germany (*Koordinationssrat der Muslime in Deutschland*, KRM) in order to fulfil a condition for recognition emphasised repeatedly by German politicians: providing the government with a central contact point for all Muslims in Germany. In some federal states, Islamic organizations have at least been recognised as religious communities (under Article 7[3] GG) and are thus entitled to provide religious education in the public schools. However, Islamic organizations are confronted with the fundamental challenge to fulfil organizational conditions that are inspired by the structures of the Christian churches and that do not go well with Islam as a, by comparison, less organized religion.

Islamic organizations in the Netherlands have responded to official requests to create a central contact point by establishing common associations as well. In 2003, Sunni organizations founded the Contact Organization for Muslims and the State (*Contactorgaan Moslims en Overheid*, CMO), while a federation of the Alevi, Shiite, and Ahmadiyya minorities established the Contact Group Islam (*Contactgroep Islam*, CGI) in 2004. Both umbrella organizations were accepted as official dialogue partners by the Minister of Integration, and both have met several times with governmental representatives. In addition to these regular consultations, however, such recognition does not involve the right to make any further legal claims. Whereas CMO and CGI were structurally funded by state in the first years of their existence, in 2011 the government decided to suspend financial support and treat them like any other ‘civil-society organization’ (Boender, 2014, p. 257) which is supposed to attract – paying – members.

## Defenders vs. challengers of the status quo: governmental actors and Islamic organizations

Since both in Germany and the Netherlands consultations between Islamic and governmental representatives during the analysed period (2006 – 2012) have particularly taken place in *national* advisory boards this study primarily accounts for this level. In the Netherlands, the Inter-Islamic Platform for Governmental Matters (*Inter-islamitisch Platform Overheidszaken*, IPO) was established to create an arena for consultations between the Dutch government and the two Islamic umbrella organizations CMO and CGI. The responsible government representative with regard to negotiations in the context of the IPO is the Minister of Integration^4^ and not the Minister of Justice,^5^ who is usually responsible for the government’s relationships with religious organizations. In Germany, the German Islam Conference (*Deutsche Islam Konferenz*, DIK) was established by Wolfgang Schäuble, then Federal Minister of the Interior, in 2006 to ‘indicate the path to a better legal and societal integration of Muslims in Germany and – where possible – to take this path’.^6^ In both countries, the Christian Democrats played the most important role on the governmental side.^7^ (Table 1).

**Table 1:** National State Actors and Advisory Boards in Germany and the Netherlands

	Germany	Netherlands
Government	Christian Democrats/Social Democrats (2005-2009) Christian Democrats/Liberals (2009-2013)	Christian Democrats/Social Democrats/ Christian Union (2007-2010) Liberals/Christian Democrats (2010-2012)
Responsible Ministers	Ministers of the Interior Wolfgang Schäuble (Christian Democrats; 2006-2009) Thomas de Maziére (Christian Democrats; 2009-2011) Hans Peter Friedrich (Christian Social Union; 2011-2013)	Ministers of Justice Ernst Hirsch Ballin (Christian Democrats; 2006-2010) Ivo Opstelten (Liberals; 2010-2012) Minister of Integration Ella Vogelaar (Social Democrats; 2007-2008) Eberhard van der Laan (Social Democrats; 2008-2010) Geerd Leers (Christian Democrats; 2010-2012)
Advisory Boards	German Islam Conference (DIK, since 2006)	Inter-Islamic Platform for Governmental Matters (IPO, since 2005) Consultative Council of Turks in the Netherlands (IOT, since 1986)

In Germany, Muslim immigrants from Turkey and their descendants form by far the largest ethnic minority today which is why the majority of Islamic organizations that established the umbrella organizations KRM in Germany are of Turkish origin. In the initial phase of the DIK, all members of KRM, as well as the Alevi minority, took part in the conference; however, as of 2010, the Islamic Council, which was no longer invited, and the Central Council, which decided to boycott the conference after complaining about missing results, ceased to participate.

In the Netherlands, the Moroccan minority group is almost as large as the Turkish one; however, the organizational landscape of Muslims is also dominated by Turkish immigrants. This is why in both countries Turkish-Islamic organizations in particular have been included in the study. Many Turkish-Islamic organizations in the Netherlands have also been members of the Consultative Council of Turks in the Netherlands (*Inspraakorgaan Turken in Nederland*, IOT), which has been part of the advisory boards system for ethnic minorities since the 1980s (Musch, 2011).

The largest Islamic organizations in these two countries are branches of the Turkish Directorate of Religious Affairs (Diyanet) which represent the official Turkish perception of laicism (Boender, 2014). After these Diyanet branches, organizations representing the Millî Görüş movement represent the second largest Islamic group in both Germany and the Netherlands. The mystical Süleymancılar movement, which, like Millî Görüş, is opposed to laicism in Turkey, was already founded in the 1920s. The Alevi minority differs from Shiites and Sunnis in many key aspects and is often not accepted as a Muslim community by other Muslims. However, as the Alevi have been involved both in the German DIK and the Dutch IPO this study has considered them, too.

In contrast to the Netherlands, two independent Muslim umbrella organizations have been established in Germany; these include not only both Sunni and Shiite Muslims but also Muslims of different national origins: the Central Council of Muslims and Germany and the Islamic Council (Musch, 2011) (Table 2).

**Table 2:** Selected Islamic Organizations in Germany and the Netherlands

National Origin	Religious Basis	Islamic Organizations Netherlands	Islamic Organizations Germany
Turkish	Sunni (Diyanet)	Foundation Turkish-Islamic Cultural Federation (STICF) Islamic Foundation in the Netherlands (ISN)	Turkish-Islamic Institution for Religion (DITIB)
Turkish	Sunni (Milli Görüş)	Dutch Islamic Federation (NIF) Milli Görüş Northern Netherlands (MGNN)	Islamic Community Milli Görüş (IGMG)
Turkish	Sunni (Süleymancılar)	Foundation Islamic Center of the Netherlands (SICN)	Organization of Islamic Cultural Centers (VIKZ)
Turkish	Alevi	Federation of the Alevi Community in the Netherlands (Hakder)	Alevi Community in Germany (AABF)
Multinational	Sunni and Shiite	–	Islamic Council for the Federal Republic of Germany (IRD) Central Council of Muslims in Germany (ZMD)

Participating in Umbrella Organizations:

Germany: DIK (Islamic): DITIB, VIKZ, AABF, Islamic Council (until 2011), Central Council (until 2011)

Netherlands: IPO (Islamic): all; IOT (Turkish): all

## Empirical analysis: How German and Dutch governmental actors and Islamic representatives view the *Integration of Islam*

Many studies have indicated an increasing *securitization* of Islam policy in Western European countries where governmental actors conceive of Muslims and Islam as a potential (terrorist) threat and, therefore, perceive the *integration of Islam* first and foremost as a means of improving public security. This has also been emphasized in studies on Germany (cf. Brunn, 2012; Halm, 2013; Teszan, 2012) as well as the Netherlands (cf. Boender, 2014; Koomen, Tillie, van Heelsum, & van Stiphout, 2013). Since researchers have already covered this aspect sufficiently, the focus of this analysis is not on this issue.

### The state, religion, and religious pluralism

In their discourses on the *integration of Islam*, representatives from Islamic organizations in Germany and the Netherlands and the two governments regularly address two general questions: how to arrange the relations between the state and the religions of the country in question and how to handle religious pluralism.

#### Governmental actors in Germany

In describing the relationship between the state and religion, German governmental actors often use terms such as ‘positive neutrality’^8^ and ‘stimulating neutrality’.^9^ Politicians in Germany do not see the state’s neutrality as a passive or indifferent attitude towards religions and religious organizations; rather, because a role for religious groups in the public sphere is encouraged, the politicians conceive of these groups as potential contact partners and express their willingness to participate in dialogue. The state is thus responsible for creating institutional conditions that will facilitate the cultivation of religion. In 2007, Minister of the Interior Schäuble noted that Germany’s ‘secular state depends on the meaningful power of religion. Accounting for the imperative of neutrality, which derives from the freedom of religion, the state cooperates with organized religious communities’.^10^

However, neutrality does not mean that the distance is equal between the state and each of the different religions that have established themselves in Germany. Instead, all three Ministers of the Interior who held this office between 2006 and 2012 referred to Christianity as a culture that has fundamentally shaped German society. In 2011, Minister de Maizière argued, ‘It is for sure that juridical neutrality is not a synonym for equidistance. This derives from the preamble of the Constitution. We are shaped by Christianity culturally, spiritually and politically’.^11^

#### Governmental actors in the Netherlands

The Dutch governmental actors refer explicitly to religions in the plural, thus emphasising the legitimacy of religious pluralism more explicitly than the German governmental actors do. Second, the Dutch authorities also speak much more often of world views, thus highlighting the equal status of both religious and secular views in public life and thereby reiterating the acceptance and support of pluralism in the Netherlands.^12^

In terms of the separation of religion and the state, neither the Dutch nor the German governmental actors imply that the two contingents should not interact. Nevertheless, the two countries differ when it comes to the governments’ motivations in approaching religions and their organizations. Whereas in Germany the governmental actors explicitly see themselves as responsible for cultivating religion, which is defined as a value in and of itself, governmental actors in the Netherlands see religious organizations, as they do other collective societal actors, as cooperative partners in implementing their policy. In 2007, the Dutch cabinet concluded that ‘[i]n the Dutch context the separation of Church and State is established in a way that enables the government to cooperate with religious-based organizations when making its policy. When doing so, the government is obliged to treat all world view communities equally according to article 1 of the constitution and not to side with a certain stream’.^13^

When it comes to responsibilities of the state regarding religious matters, government officials above all base their policy on the concept of religious *pluralism*. This is why establishing a wide range of religious interpretations within society becomes an important task of the government. In 2008, then Minister of Justice, Ernst Hirsch Ballin, argued that ‘[t]he Netherlands is characterized…by a “pluralistic cooperation”. This includes […] not interfer[ing] with the private sphere, but [the government] can provide for the opportunity of religious and world view organizations to unfold activities of general societal interest’.^14^

#### Islamic organizations in Germany

Because Islamic organizations in Germany agree with governmental actors about the important role of religion in public life, they warn explicitly of the danger of privatising religion: ‘[r]eligion must not be repelled, as this would cause more discrimination in society because pious people, religious people in society would be judged if they did something religious’.^15^ Thus, Islamic organizations do not foresee certain religious communities excluding themselves but observe a general trend towards society excluding religion from the public sphere: ‘there is a common mainstream [view] that religiosity, no matter if it’s Islamic or Christian, is not frowned upon, that just wants to repel religiosity from the public realm’.^16^

The same point was made in a press release published by KRM in 2011, in which the organization criticised the decision of the Federal Administrative Court (*Bundesverwaltungsgericht*) allowing schools under particular circumstances to forbid pupils to pray at school: ‘with this decision we run the risk of repelling not only Islam but religiosity in general more and more from the public realm’.^17^

By using such arguments, the Muslim community is able to show that the problem does not just affect Muslims, but is also an urgent matter for the Christian churches. Thus, their own experiences with the difficult position of Muslims and Islam in Germany represent a common problem for religions and religious people in general: ‘this is our general problem that we as Muslims and Christians both have to deal with. […] Actually, Churches and their representatives are our friends. They have the same interests, the same motives we have.’.^18^

Although Islamic organizations in Germany criticise the trend of privatising religion, they praise the opportunities for cooperation between religions and the government that exist in Germany. In their view, this means that religious communities are appreciated as actors who can bring important values to the society. Most representatives of Islamic organizations explicitly support the German model of cooperation between the government and religious communities. A representative from the Islamic Community Millî Görüş pointed out that ‘German State Church Law offers a great potential when it comes to the inclusion of religious communities which are – unlike the Catholic and the Protestant Churches – not established yet’.^19^

#### Islamic organizations in the Netherlands

The respective positions and patterns of argumentation of the Dutch and the German Islamic organizations differ in terms of self-portrayal. Whereas the Islamic organizations in Germany define themselves and argue as religious communities and thereby as direct counterparts of the Christian churches, the Islamic organizations in the Netherlands describe their main focus as social and cultural while downplaying their religious character. This social and cultural role not only affirms their legitimacy as societal actors along with other religious and non-religious collective interests, but it may also afford them public financial support because they fear exclusion from this benefit if the religious character of their organizations is perceived as compromising the policy of separation of religion and the state. Therefore, their representatives rarely comment on the role of religion in the public sphere. On its website, CMO complains only in general terms about ‘different negative developments resulting from erupting secularization and individualization’ without explicitly demanding a stronger role for religion in the public realm, as their German counterparts do.^20^

Islamic organizations in Germany and in the Netherlands also differ in their respective views concerning the separation of religion and the state. Unlike their German counterparts, Islamic representatives in the Netherlands express their acceptance of a general separation, including the fact that the government refrains from funding religious organizations. A representative from the CMO-member STICF argues that the ‘[s]eparation between Church and state is very important’.^21^ A representative from the Alevi organization HAKDER, a member of the umbrella organization CGI, agrees: ‘a secular state may never give money to a religious community. […] If people believe in something, they have to fund it themselves’.^22^

However, the Islamic organizations in the Netherlands demand that this policy of separation of religion and the state be applied to all religions in the same way. According to some representatives, Muslims might be at a disadvantage in this respect because the state has occasionally denied funding for their social and cultural activities based on this policy. Therefore, they insist on de facto neutrality on the part of the state when it comes to denying support to religious communities. A representative of Millî Görüş Noord Nederland (MGNN) suspects the state of favouring the Christian churches, saying, ‘the state is neutral, state and church are separated and this has to be a real fact!’^23^

### The position of Islam in society and the status of Islamic organizations

Both government representatives and Islamic organizations refer to the status of Islam in their respective receiving societies in general. Furthermore, the focus of the debate is on the character of the Islamic organizations and their role in the *integration of Islam.*

#### Governmental actors in Germany

In Germany there has been a sometimes polarised debate in the political realm over whether or not Islam ‘is a part of’ or ‘belongs to’ the country. Whereas the Ministers of the Interior Schäuble and de Maizière have supported this view, their successor Minister Friedrich has believed that ‘[t]he view that Islam is a part of Germany is a fact which cannot be proved historically’.^24^

Furthermore, governmental representatives try to counter the request for recognition made by Islamic organizations, arguing that Islam is already a recognised religion in Germany because it enjoys the freedom of religion enshrined in the German Constitution.^25^

With regard to the relationship between the German state and Islam, governmental representatives have asked how religious communities must be organized in order to serve as an official contact point, stressing that a self-definition of religious organizations as religious communities is not a sufficient condition. Minister Friedrich argued in 2012, ‘Muslims want to be treated by the state in the same way as the Christian Churches are treated. For this they need to create the organizational conditions’.^26^

German representatives at both the federal and the state levels have failed to support the recognition as religious communities or public corporations, contending that Islamic organizations have not fulfilled the necessary criteria. In 2007, then Minister of the Interior Schäuble described the Islamic organizations as ‘interest groups’ and expressed his doubts regarding their representativeness: ‘I already indicated […] that the four largest organizations have 300,000 members and that they thus only represent about ten per cent of the Muslims living in Germany’.^27^

German governmental representatives hold the Islamic organizations themselves responsible for not yet having achieved recognition. In 2008 Schäuble explained: ‘now it has become clear that the Muslims themselves have to make sure that they achieve the status of a religious community – it won’t just be put into their hands as some may have assumed’.^28^ His successor, Thomas de Maizière, also complained about the lack of ‘willingness of some Islamic organizations to adapt their organizational structures’.^29^ On the other hand, referring to the example of the Christian churches, he described the establishment of institutionalised relationships between Islamic organizations and the state as a long-term task: ‘it would be presumptuous and ahistorical to demand the establishment of institutionalised cooperation between state and Muslims overnight’.^30^

#### Governmental actors in the Netherlands

In the Netherlands, the discussion about Islam has recently been shaped by the right-wing populist Geert Wilders and his Party for Freedom (*Partij voor de Vrijheid*, PVV), which has defined Islam as an evil ideology planning to dominate and finally ‘islamicise’ the Netherlands – a threat which, in his view, needs to be combated.^31^ This view is not shared by the other parties represented in the Dutch parliament. The centre-right liberal People’s Party for Freedom and Democracy (*Volkspartij voor Vrijheid en Democratie*, VVD) and the Christian Democratic Appeal (CDA), which formed a minority coalition tolerated by the PVV between 2010 and 2012, have defined Islam as a religion that should be treated the same way as any other religion. Unlike in Germany, Christian Democratic politicians have rarely confessed to a Christian tradition of their country. In 2007, the government, then led by the Christian Democrats, emphasised instead that ‘[t]he debate [on Islam] sometimes neglects [the fact] that Islam constitutes a part of the fabric of our society, just like every other world religion in our country’.^32^

Unlike German politicians, who define freedom of religion as a *Christian* tradition, their Dutch counterparts perceive it primarily as a *Western* value. Because of this, they refer particularly to the issue of equal rights and less to a presumed Christian tradition of the Netherlands when dealing with the integration of Islam: ‘taking the growing part of the Dutch population into account which is affiliated with Islam is an expression of the Western values of freedom of faith and tolerance’.^33^

Again, in contrast to German officials, governmental representatives in the Netherlands emphasise the state’s obligation not to interfere with the internal issues of religious organizations, not only in terms of the theological issues they address but also with regard to their organizational structures. When it comes to the government’s relationship with Islamic representatives, the criteria for recognition are much less strict than those of their German counterparts. The two peak organizations, CMO and CGI, achieved recognition because they defined themselves as religious communities, because they were recognised as a religious group by other religions as well as by religious scientists and because of their representativeness. Although only a minority of Muslims living in the Netherlands are members of these two organizations, they were nevertheless recognised by the Ministry of Integration because, in the case of CMO, the majority of mosques are affiliated with them and, in the case of CGI, a wide range of Islamic streams are involved. Thus, not only does the definition of ‘representativeness’ differ from that in Germany; the requirements for representation also differ between the two peak organizations in the Netherlands. However, although CMO and CGI have been accepted as *Islamic* contact partners, government representatives emphasise that their consultations with them are primarily concerned with integration and not with religious matters.^34^

#### Islamic organizations in Germany

Islamic organizations have stressed repeatedly that Islam is a part of Germany and must be treated in the same way as other religions in general and the Christian churches in particular. In 2008, one board member of the Turkish-Islamic organization DITIB pointed out that ‘[i]n Germany we have, chronologically speaking, a Jewish-Christian-Islamic-Occidental culture, and every religion has contributed to this. This is how we will solve the whole problem. […] Then Muslims will say, “Yes, this is my culture, my country!’”^35^

However, Islamic representatives complain about the lack of political will to recognise Islam and its organizations as religious communities or public corporations. Their argument for their recognition is twofold: On the one hand, they claim to have already fulfilled the conditions of the Constitution and refer to court decisions in their favour on this issue. One representative of the Alevi organization AABF noted, ‘I think our chances [of getting recognition as a public corporation] would be very good if we went to court’.^36^ On the other hand, they demand modifications to the criteria enshrined in the church–state law which, in their view, is exclusively geared to the structure of the Christian churches and does not give organized Islam, with its different structures, the chance to meet the criteria for recognition. As a representative of the Islamic Council put it: ‘some criteria [for recognition] only make sense for the Churches’.^37^ Therefore, they warn of a de-legitimisation of the whole German church–state system because the different religious communities are not deemed equal. In 2011, KRM warned in a press release, ‘if the recognition and integration of the Islamic religious communities will not succeed, the traditional system between state and religion will lose its legitimacy and the status of the Christian Churches will be an untenable privilege for other religious communities’.^38^

The Islamic representatives also emphasise the common interests between the Islamic organizations and the churches and describe the latter as their natural allies. Comparing themselves with the Christians is also a strategy to counter state representatives that accuse the Islamic organizations of being too conservative: ‘as Muslims we are as conservative as the Catholics are’.^39^

Thus, for Islamic representatives in Germany, the issue of integrating Islam is primarily about the structural integration of their organizations. In their view, this structural integration is needed to acknowledge the fact that Islam belongs to the country. At the same time, they demand that this structural integration be accompanied by the right to self-determination as enshrined in the constitution.^40^

Unlike the German government officials, the Islamic organizations see the state as responsible for creating the conditions for their recognition. In their view, Islamic organizations have spent decades making an effort to meet those conditions.^41^ When it comes to the question of their own representativeness, KRM uses the same argument the Dutch officials relied on when they accepted CMO as a contact partner – that is, they serve to unite a vast majority of mosques.^42^

#### Islamic organizations in the Netherlands

Regarding the position of Islam in the Netherlands, Islamic representatives emphasise the separation of their religion from the state by refusing to accept any subsidies for their religious activities. In 2008, a representative of the Diyanet organization STICF stated, ‘Churches are not funded by the state and [neither are the] mosques. And we wouldn’t want that, we don’t want to be supported as a mosque; we will cover the expenses ourselves’.^43^ Nevertheless, they accuse the Dutch authorities of disfavouring Muslims in their interpretation of the separation of religion and state. In particular, they complain about state representatives who have repeatedly refused to fund their social and cultural activities, pointing to the separation of religion and the state. In the view of a representative from the CMO member SICN, this problem might be solved if ‘secularism [were] interpreted properly’.^44^ If that were the case, the Dutch authorities would realise that there is no harm in funding the comparatively dominant social and cultural activities of the Islamic organizations only. According to a STICF representative, ‘apart from being a mosque we are a societal organization. But it is very difficult to point out this difference when asking for subsidies’.^45^

With regard to the status of the umbrella group CMO, its member organizations emphasise its recognition as a consultation partner and see themselves as representing the interests of Muslims in the Netherlands and a bridge builder between the Islamic community and the Dutch state. They repeatedly point out CMO’s representativeness as a body that unites the vast majority of Islamic communities within the country. On its website, CMO also stresses its ‘practicable, open and democratic structure’,^46^ which is in clear contrast to KRM, which, in its self-understanding as a religious community, does not have to emphasise a democratic internal decision-making process.

## Discussion and conclusion

### Institutionalist and constructivist explanations

The results of this project support the benefit of disentangling institutionalist and constructivist assumptions deriving from the concept of political opportunity structures. This helps to uncover to what extent there is cross-national divergence with regard to the views on the *integration of Islam* and how far differences between the positions of governmental and Islamic actors can be observed.

First, the analysis has shown that there are indeed cross-nationally different views that can be traced back to divergent opportunity structures prevalent in the two countries – as expected by institutionalist perspectives. The German state–church regime, which provides for narrow cooperation patterns between government and religious representatives, serves as a point of reference for both governmental actors and Islamic organizations favouring the establishment of institutionalised relations. In the Netherlands, which lacks an institutionalised framework for cooperation patterns and where the focus has recently been on preserving the separation between religion and the state, the idea of deepening mutual relations is of less importance.

Opportunities deriving from the regime of religious governance are thus more significant in Germany than in the Netherlands. This is also why the German authorities are more reluctant to recognise Islamic organizations than the Dutch officials since the consequences are more expansive in Germany. On the other hand, the far-reaching opportunities deriving from the German regime of religious governance facilitate decision making on the part of (Turkish-)Islamic organizations on how they should present themselves – that is, as religious communities and, thus, as Islamic counterparts to the Christian churches. This question is more complicated for Turkish-Islamic organizations in the Netherlands, where opportunities are rarely offered by the regime of religious governance but instead by the regime of immigrant integration. Turkish-Islamic organizations in the Netherlands thus opt to walk the tightrope: While, on the one hand, they cooperate with government representatives as religious actors trying to support the integration of their religion within the country, on the other hand, they portray themselves as (ethnic) social and cultural organizations whose activities are completely separate from any religious matters. This entitles them to be part of the ethnic advisory board system and to benefit from public funding. For their German counterparts, who do not see any chance of being accepted and supported in their role as (ethnic) migrant organizations, portraying themselves as such is not an option. Interestingly, this cross-national difference holds for Islamic organizations of different streams such as Sunnis and Alevis which usually disagree and have not succeeded in establishing common peak organizations.

The different views of governmental actors in Germany and in the Netherlands regarding religious pluralism can also be traced back to divergent national regimes of religious governance. Although the patterns of cooperation between religion and the state in Germany are in principle not exclusive, it is the Christian churches in particular that have benefitted. Consequently, familiarity between the Christian churches and the German state has grown, and the state is now more likely to identify with Christianity despite all public avowals of its neutrality. In the Netherlands, creating equal opportunities for different religions and world views has been perceived as a task of the state since the years of a *pillarised* Dutch society. This is why Dutch authorities tend to argue for neither an exclusively Christian tradition nor religion as a value as such. Instead, they focus on the realisation of a pluralistic Dutch society where diverse religious and non-religious actors may coexist as equals.

However, the study has also shown that looking for cross-national differences originating from national context factors does not provide the whole picture. In contrast: In line with constructivist expectations, the analysis has revealed divergences in the framing strategies employed by government representatives and Islamic organizations that particular derive from a clash of interests in the discourse. As representatives of a religion that has immigrated and is therefore still (perceived as being) foreign in Western European countries, Islamic organizations inevitably play the role of, and argue as, challengers of the status quo, while their official government contact partners play the role of its defenders. In Germany, this is true primarily of questions regarding religious pluralism and of the way in which Islam is framed as a *case*. Government officials seek to present Islam as a particular religion that does not ‘belong’ to Germany or at least will never have the relevance for the country as Christianity has had, and which might be incompatible with German cultural values (which is a point that other researchers such as Brunn (2012), Halm (2013), and Teszan (2012) have also underlined). By this the German government refers to the frame of *treating unequal things unequally* emphasizing why (organized) Islam cannot be granted the same status as Christianity and the churches. Government officials’ references to structural shortcomings of the Islamic organizations, as compared with the Christian churches, describing them as interest groups instead of religious communities is also in line with the aim to highlight differences and, consequently, to legitimise unequal treatment. Islamic representatives argue for the exact opposite. By framing Islam as a direct counterpart to Christianity and themselves as appropriately organized Islamic counterparts to the Christian churches, they imply that their official recognition as religious communities is a logical step towards achieving *equal treatment*. This strategy is also reflected by statements that depict the Islamic organizations as natural allies of the Churches that may also speak for people with a Christian religious affiliation.

Different framing strategies used by government and Islamic representatives can also be observed in the Netherlands. Unlike in Germany they do not disagree on the status of Islam in the country but only on the character of the Islamic organizations. Whereas governmental representatives accept Islam being a religion like any other that deserves *equal treatment* this does not hold for the Islamic organizations when it comes to supporting them financially. Pointing to the religious character of these organizations governmental representatives refuse to support them with public funds as it might contradict church–state separation. In their view, Islamic organizations are different from other ‘societal organizations’ and, consequently, may be treated unequally. In contrast, the organizations try to frame themselves as primarily social and cultural organizations and consider their request for public financial support to be legitimate. By framing their organizations and their activities as mainly non-religious, they hope to convince the state that *equal treatment* with other societal organizations, thus public funding is a possible, even necessary, option. However, Islamic organizations in the Netherlands pursue a two-track strategy when it comes to classifying themselves. Their self-portrayal as primarily sociocultural does not prevent them from simultaneously describing themselves as Islamic counterparts to the Christian churches. The organizations depend on this strategic move when making use of their pattern of argumentation for *equal treatment*. In recent years, government representatives have claimed that the organizations themselves should make more of an effort to improve the integration of Muslims living in the country. The organizations have responded by framing consultations within the IPO as a dialogue between religion and the state, comparing the situation to the contacts between the state and the Christian churches. This view clearly contradicts that of the Dutch government, which perceives such consultations as negotiations about integration policies. Interestingly, government actors pursue this same two-track framing strategy when defining the Islamic organizations as religious organizations to refuse certain demands (i.e. funding) and as social and cultural organizations when trying to exploit them for their political agenda (i.e. integration policy).

All in all, the findings of this study support the conclusion that when it comes to positions put forward by governmental representatives, their role as defenders of a status quo seems to outbalance the impact of the particular national context they are located in. When push comes to shove or, put differently, when positions are to be translated into concrete measures, governments seem to be pulling back to their *treating unequal things unequally* frame and refrain from granting (organized) Islam the equal rights that the respective national regimes of religious governances would actually allow for. In contrast, it rather seems to be representatives from Islamic organizations who (implicitly) refer to opportunities which the respective national context factors provide.

### The *Integration of Islam* in Western European countries

The findings of this study also allow for implications with regard to the integration of Muslims and Islam in Europe. Although the principle of religious pluralism prevails in all Western societies, the self-understanding as a presumably ‘Christian country’ is still alive in some countries (Germany) more than in others (the Netherlands). Therefore, the discourse on the *integration of Islam* is not least a conflict between two principles: the neutrality of the state towards religions (and world views) and the Christian heritage and imprinting of the society. Particularly in Germany, the fact that many (conservative) governmental representatives deny that Islam is ‘a part of Germany’ impedes the integration of the religion, although the basis established by the country’s regime of religious governance is actually promising. This also explains why there has been only slow progress when it comes to establishing Islamic education at public schools or an Islamic welfare organization in spite of a constitution that provides for quite favourable conditions. In the Netherlands, where the regime of immigrant integration (still) supplies opportunities for immigrants, this does not hold to the extent for the country’s regime of religious governance. Although it shows more openness towards religious pluralism, the increasingly laicist interpretation of the separation between religion and the state has limited chances of Islamic organizations to be supported or funded by the state. Therefore, Dutch authorities have rather been hesitant about seriously cooperating with Islamic organizations – and not only exploiting them to achieve their goals in integration policy. Consequently, Islamic organizations have met more and more difficulties when trying to establish Islamic schools or commit themselves in the welfare sectors.

Whereas Islamic representatives appear to have internalized national church-state traditions quite successfully, governmental actors seem to deny their own country’s traditions by applying strategic framing. However, the *integration of Islam* in Western Europe might benefit if state representatives took the concept of equal treatment of religions to heart when it comes to Islam and thereby empowered Islamic organizations to meet the demands for their recognition and participation according to national church–state regimes.

## Footnotes

1 Studying protest behaviour in American cities Eisinger (1973, p. 11) referred already in the 1970s to the “complex interrelationship between political environment variables on the one hand and political behavior on the other”.

2 According to Article 140 of the German Basic Law (*Grundgesetz*, GG), Articles 136 and 137 of the Weimar Constitution *(Weimarer Reichsverfassung*, WRV) were incorporated into the area of application of the GG. Article 137(5) of the WRV establishes the status of a public corporation.

3 translation by the author

4 as of 2010 the Minister of Immigration and Asylum

5 as of 2010 the Minister of Justice and Security

6 http://www.deutsche-islam-konferenz.de/DIK/DE/DIK/1UeberDIK/DIK06-09/Rueckschau/rueckschau-node.html; all German and Dutch primary sources translated by the author.

7 Consequently, this study only allows conclusions regarding views of government politicians that are members of Christian Democratic or conservative parties. Future studies should also focus on arguments raised by liberal of leftist parties in order to uncover to what extent partisanship might be of relevance in this context.

8 http://www.deutsche-islam-konferenz.de/DIK/DE/Service/Bottom/RedenInterviews/Reden/20090306-theologisches-forum.html.

9 https://www.tagesspiegel.de/politik/innenminister-friedrich-im-interview-der-papst-spricht-als-staatsoberhaupt/4628810.html.

10 http://www.deutsche-islam-konferenz.de/DIK/DE/Service/Bottom/RedenInterviews/Reden/20090306-theologisches-forum.html.

11 https://www.tagesspiegel.de/politik/innenminister-friedrich-im-interview-der-papst-spricht-als-staatsoberhaupt/4628810.html; http://www.deutsche-islam-konferenz.de/SharedDocs/Anlagen/DIK/DE/Downloads/Sonstiges/Dokumentation%20IRU-Tagung%202011.pdf?__blob=publicationFile.

12 https://www.wrr.nl/binaries/wrr/documenten/rapporten/2006/04/18/dynamiek-in-islamitisch-activisme.-aanknopingspunten-voor-democratisering-en-mensenrechten/R073-Dynamiek-islamitisch-activisme.pdf.

13 https://www.wrr.nl/binaries/wrr/documenten/rapporten/2006/04/18/dynamiek-in-islamitisch-activisme.-aanknopingspunten-voor-democratisering-en-mensenrechten/R073-Dynamiek-islamitisch-activisme.pdf.

14 https://www.trouw.nl/home/hirsch-ballin-religie-en-samenleving-een-geheel~ae474782/.

15 Verband der Islamischen Kulturzentren [VIKZ] interview, 6/24/2008

16 Zentralrat der Muslime in Deutschland [ZMD] interview, 6/9/2008

17 http://www.vikz.de/index.php/pressemitteilungen/items/krm-mahnt-vor-dem-herausdraengen-der-religion-aus-dem-oeffentlichen-raum.html.

18 ZMD interview, 6/9/2008

19 Islamische Gemeinschaft Millî Görüş [IGMG] interview, 6/17/2008

20 http://cmoweb.nl/ontstaansgeschiedenis/.

21 Stichting Turks-Islamitisch Culturele Federatie [STICF] interview, 11/21/2008

22 HAKDER interview, 10/13/2008

23 Millî Görüş Noord Nederland [MGNN] interview, 11/10/2008

24 http://www.deutsche-islam-konferenz.de/DIK/DE/Service/Bottom/RedenInterviews/Reden/20110728-ministerrede-plenum2011.html.

25 https://www.tagesspiegel.de/politik/innenminister-friedrich-im-interview-der-papst-spricht-als-staatsoberhaupt/4628810.html.

26 http://www.deutsche-islam-konferenz.de/DIK/DE/Service/Bottom/RedenInterviews/Reden/20110728-ministerrede-plenum2011.html.

27 http://www.deutsche-islam-konferenz.de/DIK/DE/Service/Bottom/RedenInterviews/Interviews/20070422-faz-net.html.

28 https://www.welt.de/politik/article844528/Schaeuble-sieht-kein-Monopol-der-Muslim-Verbaende.html.

29 https://www.tagesspiegel.de/politik/innenminister-friedrich-im-interview-der-papst-spricht-als-staatsoberhaupt/4628810.html.

30 https://www.tagesspiegel.de/politik/innenminister-friedrich-im-interview-der-papst-spricht-als-staatsoberhaupt/4628810.html.

31 https://www.tweedekamer.nl/downloads/document?id=42d4fd0b-5e7b-407a-bb2e-716728a08dc2&title=Antwoord%20op%20vragen%20van%20het%20lid%20Fritsma%20over%20de%20start%20van%20de%20bouw%20van%20de%20Westermoskee.doc.

32 https://www.wrr.nl/binaries/wrr/documenten/rapporten/2006/04/18/dynamiek-in-islamitisch-activisme.-aanknopingspunten-voor-democratisering-en-mensenrechten/R073-Dynamiek-islamitisch-activisme.pdf.

33 https://www.tweedekamer.nl/downloads/document?id=e765b35a-0e36-4a4c-8319-63da8f68950e&title=De%20wasruimte%20op%20een%20hogeschool.pdf.

34 https://www.wrr.nl/binaries/wrr/documenten/rapporten/2006/04/18/dynamiek-in-islamitisch-activisme.-aanknopingspunten-voor-democratisering-en-mensenrechten/R073-Dynamiek-islamitisch-activisme.pdf. This distinction becomes even more apparent given the fact that the Minister of Integration is the responsible actor in these discourses, whereas consultations between state representatives and the Christian churches are the responsibility of the Minister of Justice.

35 Türkisch-Islamische Union der Anstalt für Religion [DITIB] interview, 6/14/2008.

36 Alevitische Gemeinde in Deutschland [AABF] interview, 5/26/2008.

37 Islamic Council interview, 6/12/2008

38 http://islam.de/18957.

39 VIKZ interview, 6/24/2008.

40 In a press release of 2010, KRM stated that “Islam is a part of Germany. [It’s structural integration] can only be achieved if the participation of the Islamic religious communities and their right of self-determination [are] guaranteed as... demanded by the Constitution”, http://islam.de/16614.

41 VIKZ interview, 6/24/2008

42 http://muhammad.islam.de/6833.

43 STICF interview, 11/21/2008

44 SICN interview, 12/03/2008

45 STICF interview, 11/21/2008

46 http://cmoweb.nl/ontstaansgeschiedenis/.
